# Oscillometry in Chronic Obstructive Lung Disease: In vitro and in vivo evaluation of the impulse oscillometry and tremoflo devices

**DOI:** 10.1038/s41598-019-48039-x

**Published:** 2019-08-12

**Authors:** Lennart K. A. Lundblad, Ruzica Miletic, Eeva Piitulainen, Per Wollmer

**Affiliations:** 1 Meakins-Christie Laboratories, McGill University, Montréal, QC Canada; 2 Department of Biomedical Science, Malmö University, Malmö, Sweden; 3 Department of Translational Medicine, Lund University, Malmö, Sweden; 4 Department of Respiratory Medicine and Allergology, Lund University, Malmö, Sweden; 5 Thorasys Thoracic Medical Equipment Inc., Montréal, QC Canada

**Keywords:** Laboratory techniques and procedures, Respiratory signs and symptoms

## Abstract

Impedance, or oscillometry, measurements of the respiratory system can generate information about the function of the respiratory system not possible with traditional spirometry. There are currently several instruments on the market using different perturbations. We have compared a new respiratory oscillometry instrument, the tremoflo, with Impulse Oscillometry (IOS). Patients with a physician’s diagnosis of chronic obstructive lung disease (COPD) and healthy subjects were recruited. They underwent assessment of respiratory function with oscillometry using the IOS and tremoflo devices and the resulting impedance data from the two methods were compared. The two devices were also tested against a reference respiratory phantom with variable resistances. Whereas both devices detected impairments in the patients’ lung function commensurate with small airways pathology, the tremoflo appeared to be more sensitive than the IOS. We found systematic differences between the two instruments especially for reactance measurements where the area over the reactance curve (AX) was significantly lower with the IOS compared with the tremoflo (p < 0.001). Moreover, the agreement between the two devices was reduced with increasing severity of the disease as determined with a Bland-Altman test. Testing both instruments against a respiratory phantom unit confirmed that the resistance measured by the tremoflo compares closely with the known resistance of test loads, whereas the IOS’ resistance correlated with a test load of 0.19, kPa.s.L^−1^ at higher loads it deviated significantly from the known resistance (p < 0.0028). We conclude that the absolute values measured with the two devices may not be directly comparable and suggest that differences in the calibration procedures might account for the differences.

## Introduction

The diagnosis of chronic obstructive pulmonary disease depends to a large degree on pulmonary function tests and the Global Initiative on Chronic Obstructive Lung Disease (GOLD) standard^1^ is based on the assessment of forced exhalation maneuvers by the patient. Forced exhalation maneuvers while being relatively simple to perform also have some in-built limitations^2^. Some patients cannot perform the necessary exhalation maneuver due to e.g. disease or general weakness. The physiological background of the flow-volume curve is highly complex^2^. Because lung diseases tend to reside in different compartments of the airway tree and the lung it would make sense to establish techniques that would allow for a more precise evaluation of lung function^3^. COPD is a multifaceted condition that manifests in different parts of the lungs, central vs. peripheral and that can have several causes that can lead to different manifestations and possibly to alternatives in terms of treatment^4^. It is therefore important to gain access to lung function data that are precise, repeatable and unbiased. The oscillation technique has been used extensively in animal laboratories and we have accumulated a wealth of knowledge and understanding about the interpretations of broadband oscillations from studies in animal models of lung disease^5–9^. We have also previously highlighted the need for advanced techniques to assess the severity of COPD^10^. Subsequently we have gained extensive experience from using the oscillation technique experimentally in the clinical setting^6,11–15^, demonstrating that the oscillation technique can help understanding the intricacies of lung function beyond what is possible to infer from traditional spirometry. The recently commercialized device tremoflo^16^ can assess respiratory impedance with a broadband volume perturbation, whereas another common device uses impulse oscillometry, Jaeger Masterscreen, (IOS) delivers a single square wave of a single frequency and its harmonics^17^. Differences between IOS and tremoflo were recently reported in children with asthma but it was not determined what might be the cause of the differences^18^ differences between devices was also reported in healthy subjects in another recent study^19^. In subjects with COPD, the main findings in oscillometry are an increase in Resistance and a reduction of Reactance at the lower frequencies, and an increase of the area under the curve of Reactance^11^. While both devices can detect alterations in lung function, we wanted to elucidate if the fundamental differences in the perturbation signal (single pulse v.s. a broadband sine-wave composite signal) might yield different results with the two systems. To address this question, we contrasted measurements of input impedance obtained with IOS and tremoflo. In order to obtain a material with a large range of impedance, we recruited healthy subjects as well as patients with COPD. We also constructed a lung phantom with which we made comparisons at different impedances with the two instruments.

## Methods

### Study subjects

Subjects with COPD of both sexes were recruited from patients attending the Dept. of Respiratory Medicine and referred for pulmonary function tests at the Department of Clinical Physiology, Skåne University Hospital, Malmö. All methods used and measurements performed were in accordance with relevant guidelines and regulations in effect at the time of the study. Inclusion criterion was a clinical suspicion of COPD. Exclusion criterion was inability to perform spirometry. The patients underwent spirometry according to the American Thoracic Society/European Thoracic Society standard^20–22^ and all patients with COPD were diagnosed using the GOLD standard^23^. The severity of airflow limitation was determined according to GOLD. The oscillation technique measurements were made according to the standards adopted by the European Respiratory Society^24^. The healthy volunteers, mainly hospital staff, were recruited in the same region as the patients. All were free from symptoms of respiratory disease and all were non-smokers. Spirometry was not performed in the normal subjects. If any of these subjects has sub-clinical lung disease, this would not affect the comparison of respiratory mechanics as measured by the two instruments.

### Pulmonary function tests (PFT)

Vital capacity (*VC*), total lung capacity (*TLC*), functional residual capacity (*FRC*), residual volume (*RV*) and forced expiratory volume in one second (*FEV*_1_) were measured with a MasterScreen body plethysmograph (Erich Jaeger, Würzburg, Germany). Diffusing capacity for CO (*D*_*L*,*CO*_) was measured with a MasterScreen PFT (Erich Jaeger, Würzburg, Germany). The equipment was calibrated daily according to the instructions from the manufacturer. Spirometry data are also presented as per cent of predicted as indicated e.g. *FEV*_1_%_*pred*_^25,26^. Next, the oscillation technique was used to calculate respiratory impedance using two different devices with different modes of operation and characteristics; tremoflo (Thorasys Inc. Montreal, QC) and MasterScreen Impulse Oscillometry (IOS) (Erich Jaeger, Würzburg, Germany).

### Oscillometry measurements

The tremoflo was set to deliver a 16 second volume perturbation consisting of 9 superimposed sine waves (5, 11, 13, 17, 19, 23, 29, 31 and 37 Hz) the power of the signals was inversely scaled with frequency with 5 Hz having the highest power. The tremoflo was fitted with a SureGard filter (Bird Healthcare, Melbourne, Australia) with a 99.99% bacterial/viral efficiency at a flow rate of 720 L/min removing all pollutants down to 0.027 μm. The filter has an inline dead space of 50 mL. The patient end of the filter is fitted with an elliptical opening, reducing the risk for leaks. During the measurement the patient was sitting straight up, supporting the cheeks with their hands and instructed to breathe normally while the operator triggered the oscillatory flow. The measurement was repeated three times and the average was used in the analysis.

The IOS delivers a single square-wave with a basic frequency of 5 Hz every 0.2 second, but also containing the multiple harmonics of 5 Hz (i.e. 10, 15, 20, 25, 30 and 35 Hz)^17^. The IOS was fitted with a bacterial filter similar to above.

We collected the data from the real and imaginary parts of the impedance spectrum and for the comparison we used the 5 Hz as the lowest frequency signal from both devices and the 20 Hz from the IOS and the 19 Hz from the tremoflo to represent the high frequency signal. The resonant frequency (*f*_*res*_) was calculated by the software in each device and represents the point at which the imaginary part of impedance crosses the abscissa, i.e. the point where the impedance is described by the real part only. The frequency dependence of Resistance was calculated by the manufacturers’ software as the difference between Resistance at 5 Hz (R5) and either Resistance at 19 Hz (R19) (tremoflo) or Resistance at 20 Hz (R20) (IOS) and is reported as R_5-19/20_. The reactance area (AX) was also calculated by the manufacturers’ software as the integral of the reactance curve from 5 Hz to *f*_*res*_.

All measurements in the patients were done 10 minutes after an inhalation of 0.5 mg × 3 terbutaline sulphate (Bricanyl Turbuhaler, AstraZeneca AB, Sweden), as the diagnosis of COPD is based on post-bronchodilation measurements. The healthy subjects did not receive bronchodilator.

### Measurement validation

Mechanical test loads, phantoms, were constructed to model the human respiratory system and to approximately include the impedance range of the patients. The analogs were, elastance component: a rigid glass bottle 5 L, one inertance component: a cylindrical tube (215 mm long and 24 mm diameter) and two mesh resistors (0.19 and 0.49 kPa.s.L^−1^, and the resistors in series yielding 0.68 kPa.s.L^−1^). The components were assembled to represent a respiratory system with the resistor connecting to the inertance tube which in turn connected to the elastance glass bottle. The resistor was connected to the IOS or tremoflo via a regular bacterial filter used with the patients. After an initial experiment we realized that adiabatic compression was affecting the result. To correct for adiabatic gas compression, we then filled the 5 L glass bottle with steel wool. Thanks to thermal absorption by the steel wool this approach created the condition of isothermal gas compression in the phantom.

The same perturbations used with the study subjects were then delivered to the test load 3 times for each test load and for each of the tremoflo and IOS. The SEM calculated at 5 Hz ranged from 0.038 for 0.19 kPa.s.L^−1^ to 0.054 kPa.s.L^−1^ at 0.69 kPa.s.L^−1^. At 37 Hz the SEM was 0.005 at 0.19 kPa.s.L^−1^ and 0.005 at 0.68 kPa.s.L^−1^.

### Statistics

The variables from the phantom measurements IOS and the tremoflo were compared using paired t-test. Differences between patients and normal subjects were compared using Mann-Whitney test. The Bland-Altman plots were fitted with a straight line and the regression coefficients were calculated. GraphPad Prism 8.1.1 was used for all calculations. Differences were considered significant at p < 0.05.

### Ethics approval and consent to participate

Ethical approval for the study was obtained from the Ethics Committee at Malmö University (approval number: HS60-2015/223:9) and informed consent from the participants was obtained.

## Results

### Patient demographics

The demographics of the patients is shown in Table 1. The patients were all diagnosed with various degrees of COPD (six mild, seven moderate and three severe) according to the GOLD standard^27^. The healthy subjects did not undergo any PFT.

**Table 1:** Participants’ demographics.

	Healthy subjects	Patients
N (men/women)	10 (2/8)	16 (9/7)
Age (y)	35.5 ± 13.7	54.9 ± 17.0**
Height (m)	1.69 ± 0.10	1.72 ± 0.10
Weight (kg)	68.2 ± 10.5	82.7 ± 20.1*
VC (%pred.)		100.3 ± 19.2
FEV_1_ (%pred.)		75.2 ± 19.8
FEV_1_/VC		0.61 ± 0.13
D_L.CO_ (%pred.)		80.5 ± 19.8

Mean ± SD. *p < 0.05, **p < 0.01, unpaired, two-tailed t-test.

### Contrasting impedance measurements from tremoflo and IOS

**Figure 1:**
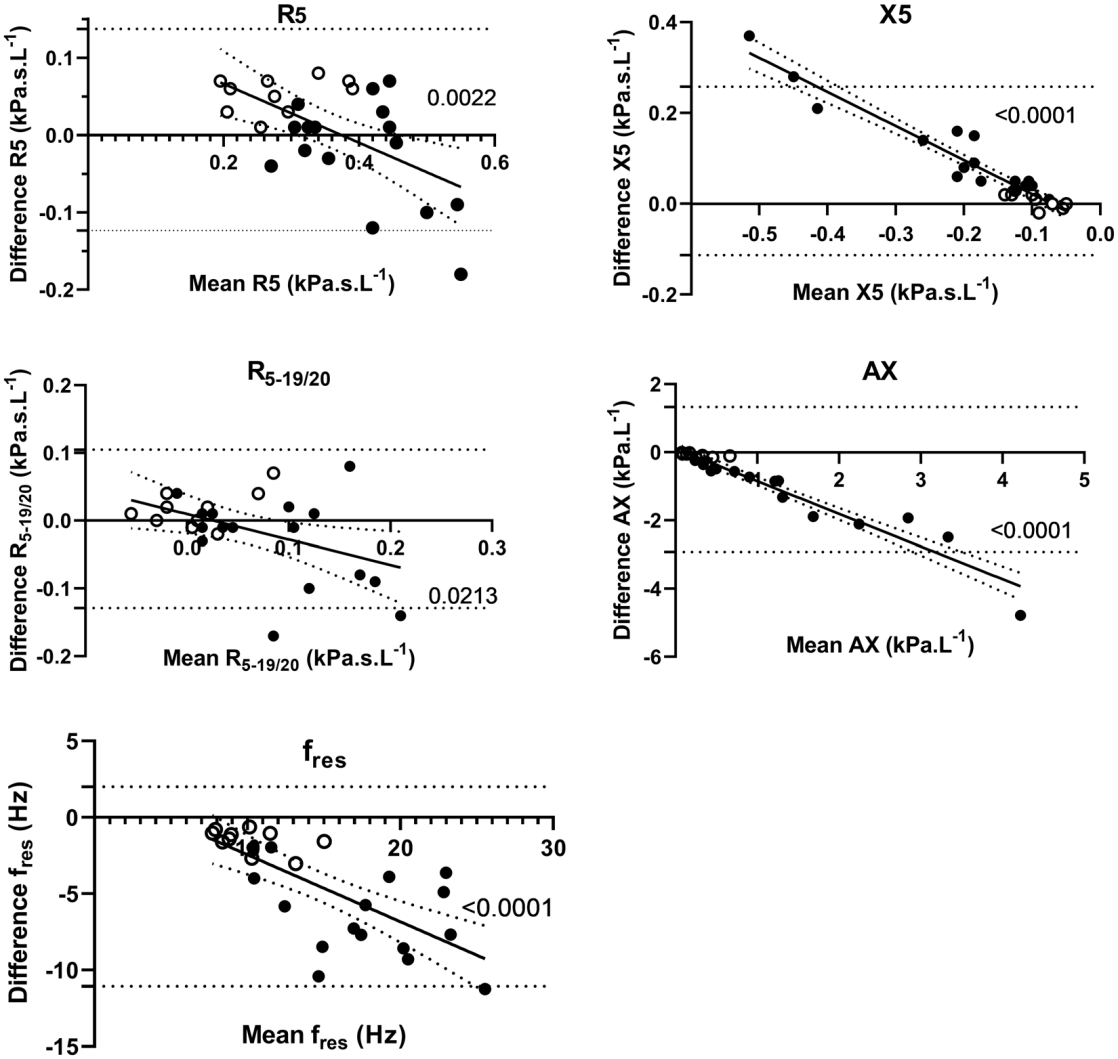
Bland-Altman plots of R5, X5, R_5-19/20_, AX, and f_res_ data obtained with IOS and tremoflo in COPD patients (solid symbols) and healthy control subjects (open symbols). A regression line was fitted to the data and the regression coefficient was calculated. The regression coefficients and associated p-values for the respective variables are; R5: r = −0.57; p = 0.0022; X5: r = −0.96; p = 0.0001; R_5-19/20_: r = −0.45; p = 0.0213 f_res_: r = −0.72; p = 0.00004; and AX: r = −0.96; p = 0.0001.

Bland-Altman plots of the measurements with IOS and the tremoflo devices are shown in Fig. 1. The most striking feature is that the bias is not constant over the range of observations as shown by the highly significant slopes of the linear curve fits. In healthy subjects, the offset is small for X5, R_5-19/20_, f_res_ and AX, whereas R5 shows consistently higher values for IOS than for tremoflo. In the patient group, the IOS instead shows lower values than tremoflo for R5. In the reactance variables, there are substantial differences between the devices, IOS showing less abnormal values, especially in the most affected patients.

**Figure 2:**
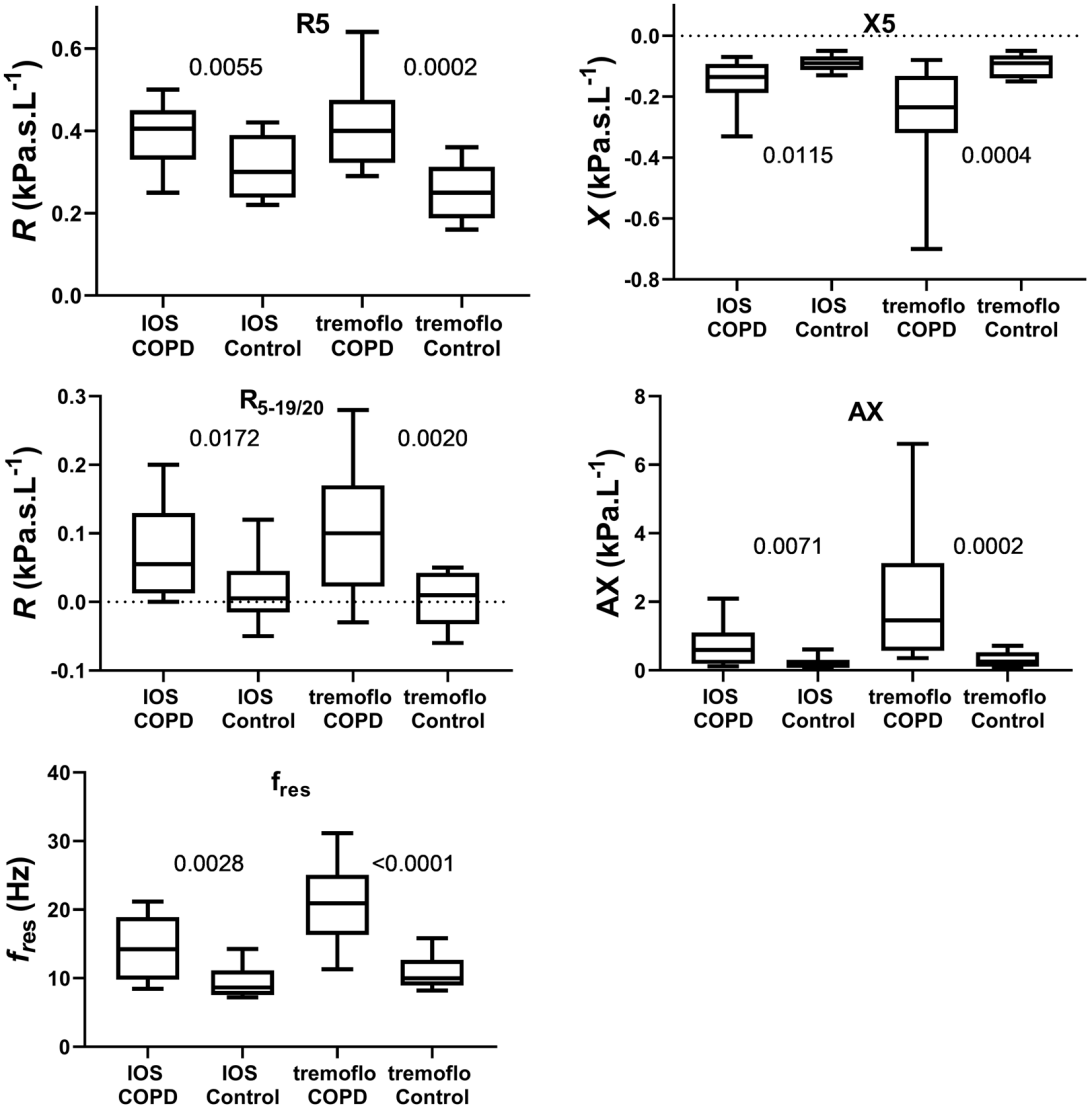
Box plots of absolute values of R5, X5, R_5-19/20_, AX, and f_res_ data obtained with IOS and tremoflo in COPD patients and healthy control subjects. Exact p-values are shown in the graphs denoting significant differences between groups of COPD and Controls for each device.

Even if the purpose of this study was to compare devices rather than patient groups, the data can be used to illustrate possible consequences for clinical studies, as illustrated in Fig. 2. These box plots clearly show that the differences observed between the healthy subjects and the patients are smaller when using the IOS than when using the tremoflo. In Table 2 it is shown that there are significant differences between the IOS and the tremoflo for healthy subjects (R5 and R20), COPD (X5, f_res_, and AX); if all subjects are averaged irrespectively of medical condition there are significant differences between the devices (X5, f_res_, and AX).

**Table 2:** Results of measurements with oscillometry.

Variable	Instrument	All subjects	COPD	Healthy subjects
R5 (kPa.s.L^−1^)	IOS	0.36 ± 0.09	0.39 ± 0.08	0.31 ± 0.08***
	tremoflo	0.35 ± 0.12	0.41 ± 0.11	0.26 ± 0.07
R20 (kPa.s.L^−1^)	IOS	0.29 ± 0.07	0.30 ± 0.08	0.40 ± 0.05***
R19 (kPa.s.L^−1^)	tremoflo	0.29 ± 0.06	0.31 ± 0.06	0.25 ± 0.04
R5-R20 (kPa.s.L^−1^)	IOS	0.05 ± 0.06	0.08 ± 0.06	0.02 ± 0.05
R5-R19 (kPa.s.L^−1^)	tremoflo	0.06 ± 0.09	0.10 ± 0.09	0.00 ± 0.04
X5 (kPa.s.L^−1^)	IOS	−0.13 ± 0.08***	−0.15 ± 0.09***	−0.09 ± 0.03
	tremoflo	−0.20 ± 0.17	−0.27 ± 0.18	−0.10 ± 0.04
f_res_ (Hz)	IOS	12.5 ± 4.4***	14.4 ± 4.4***	9.4 ± 2.3***
	tremoflo	17.0 ± 6.7	20.8 ± 5.6	10.9 ± 2.6**
AX (kPa.s.L^−1^)	IOS	0.55 ± 0.59***	0.75 ± 0.66***	0.21 ± 0.17
	tremoflo	1.34 ± 1.62	1.99 ± 1.78	0.31 ± 0.22

Mean ± SD. Significant differences between IOS and tremoflo: **p < 0.01, ***p < 0.001, paired, two-tailed t-test.

### Contrasting phantom measurements from tremoflo and IOS

**Figure 3:**
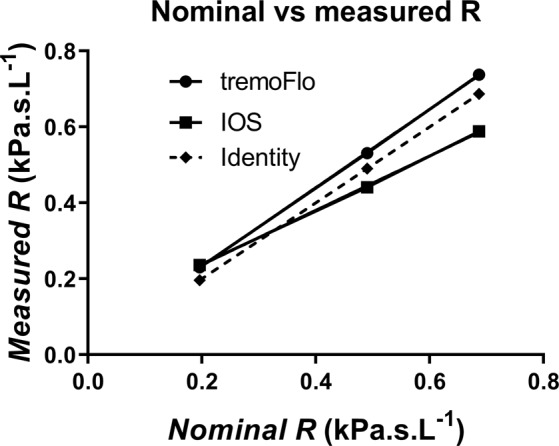
Nominal vs. measured R using calibrated resistors of 0.19, 0.49 and 0.68 kPa.s.L^−1^. Dashed line represents the theoretical line of identity if a device correctly captured the resistance value. The slopes of the tremoflo vs. IOS are significantly different (p = 0.003), the IOS slope is also significantly different from the theoretical line of identity (p = 0.0028), whereas the tremoflo slope is not significantly different from the theoretical line of identity (p = 0.057).

To verify that the two devices, tremoflo and IOS, were giving comparable results, we designed an experiment where different test loads were measured. Two experiments were performed. In the first we determined if the two devices were linear with respect to known resistance test loads at 5 Hz. Figure 3 shows the measured resistance of the test loads at 0.19, 0.49 and 0.68 kPa.s.L^−1^. Both devices behaved in a linear way but compared with the theoretical line of identity (where both measured and nominal resistances should be identical) we discovered that the IOS rendered a regression line that was significantly different from the theoretical resistance (p < 0.0028). The tremoflo, on the other hand, rendered a line that was not significantly different from the theoretical line of regression determined from the values of the test resistive loads used. The slopes of the two devices were also significantly different (p < 0.003).

**Figure 4:**
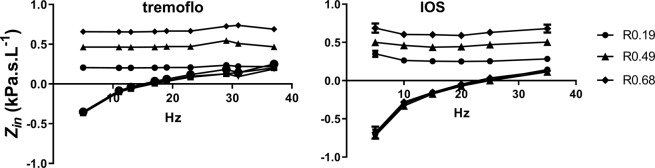
Impedance plots from phantom measurements. A glass bottle of 5 L filled with steel wool was connected to a pipe (215 mm long and 24 mm diameter) and resistors 0.19, 0.49 and 0.68 kPa.s.L^−1^. The pipe was connected to either the tremoflo or the IOS and the same perturbations as used in the patients were triggered to measure the impedance.

In a second experiment we used the three different resistive test loads in series with an inertance compartment and an elastance compartment. In Fig. 4 the impedance of the test loads at 5 L show that the 0.19, 0.49, and 0.69 kPa.s.L^−1^ were satisfactorily measured by the tremoflo throughout the frequency spectrum with little to no frequency dependence. The IOS, on the other hand, demonstrated some frequency dependence with 0.19 and 0.49 kPa.s.L^−1^ as well as with the 0.69 kPa.s.L^−1^ test load.

## Discussion

The purpose of this study was to compare the IOS and tremoflo devices in subjects with varying degree of abnormalities in lung mechanics. A salient feature of the study is that the bias of the measurements is not constant over the range of abnormalities studied. Previous studies comparing oscillation technique devices have also demonstrated inter-device differences^28,29^ thus our study further emphasizes the difficulty comparing data between oscillation technique devices.

While both the tremoflo and the IOS are based on the theory of how parameters of lung function vary with frequency, it is important to understand the differences between the two devices in terms of how they operate and how the perturbations are constructed and interpreted. The tremoflo uses a well-defined set of mutually prime frequencies ranging from 5 to 37 Hz to generate the volume perturbations used in the measurements. Furthermore, to deliver equal power at all frequencies the amplitudes of the individual frequencies are inversely related to the frequency. The result is the familiar impedance spectrum of resistance and reactance as functions of frequency, allowing for further analysis^16,28^. The perturbation is generated by an oscillating screen through which the subject is breathing also allowing for direct measurement of e.g. tidal volume. In contrast, the IOS generates a basic frequency of 5 Hz delivered as a square wave by a loudspeaker delivering impulses into the airstream of the subject^17^. This approach also generates the harmonics based on the 5 Hz signal; thus, these harmonics are not part of the perturbation signal but are the result of the way the signal is generated. Hence it is possible that differences in the engineering contributes to the differences in impedance found in this study.

While IOS and tremoflo both measure lung mechanics via applying an external signal, rather than relying on the spontaneous breathing of the patient, the theories and construction of the two devices are different. The difference is linear suggesting a systematic difference between the devices which could be explained by a slight difference in the calibration. While one might speculate that, from a theoretical point-of-view, that one device should generate higher quality measurements, this is impossible to know without a head-to-head comparison. At any rate, it is important that devices are compared with one another under realistic conditions in human subjects as well as in reference test loads such as a lung phantom.

The demographics of the COPD patients and the control subjects were significantly different in terms of age and weight (Table 1). The purpose of this study was to contrast measurements of respiratory mechanics made with two different instruments, rather than to compare patients with COPD to normal subjects. Thus, it is important to obtain a large range of subjects with different lung function whereas age and weight are less important. Nonetheless, this is potential limitation of the study that we must acknowledge. Furthermore, the number of subjects is limited and there are few patients with severe COPD. Another limitation of the study is that we do not have any pre-bronchodilator data, hence it is impossible to elucidate the reversibility in the patients. On the other hand, the diagnosis of COPD is based on post-bronchodilator spirometry according to GOLD^27^. If anything, our data would suggest that without bronchodilation the differences between the devices might have been even bigger.

An important aspect comparing two devices purporting to measure the same thing is to determine if they really do. To elucidate if the two techniques are statistically different, we constructed Bland-Altman plots which illustrate the difference in measured variables; in this case the various variables extracted from the impedance calculations (Fig. 2). We found the largest differences in X5, AX, and *f*_res_. An interesting observation is that the agreement between the two devices is reduced when the severity of the disease increases as judged from increasing resistance and progressively abnormal reactance measurements. It is obvious that subjects with a diagnosis of COPD tended to be more variable than those of our healthy control subjects and driving the slope of the plot. This means that it would be difficult to compare the absolute values measured with the different devices. In theory, it might be possible to derive correction factors from the relationships shown in Fig. 1, but that would require a larger patient material. The observation that the COPD patients demonstrated larger variance than healthy is not surprising and has been demonstrated previously using IOS^30^. It is important to note that despite the inter-device differences we discovered, both devices were able to detect differences between controls and COPD patients. However, the broadband perturbation generated by tremoflo demonstrated a larger difference between the groups in all variables studied. This might suggest that fewer subjects would be needed in e.g. a clinical study using the tremoflo than the IOS.

While the clinical data could be interpreted in a way suggesting that the tremoflo can detect a greater variability and maybe being more sensitive, this did not make much sense to us. Why would a lung appear stiffer or have higher resistance in the patients with one device while healthy subjects did not demonstrate any inter-device differences? We decided to address this conundrum by testing the two devices against a mock set-up consisting of a resistor connected in series to a pipe and a glass bottle filled with steel wool where the pipe would represent the inertance and the glass bottle the elastance compartment respectively. This revealed a systematic, linear difference between the two devices (Fig. 3). We speculate that this could be explained by a slight difference in calibration with the two devices.

Both devices were calibrated with factory supplied test resistive loads and during the calibration we used the procedures as recommended in the respective manuals. The calibration resistive loads were 0.19 kPa.s.L^−1^ for tremoflo and 0.2 kPa.s.L^−1^ for IOS respectively. The observed range of resistance in our group of subjects ranged from about 0.16 to 0.65 kPa.s.L^−1^, hence within the range of the instruments. In severe cases of bronchial obstruction, the range would likely be much larger. The analysis of the test resistors, without any compliance compartment, show that the IOS returned a slightly higher than expected resistance value at 0.19 kPa.s.L^−1^ but returned systematically lower than the nominal values of the test loads at 0.49 kPa.s.L^−1^ and 0.69 kPa.s.L^−1^. The tremoflo, on the other hand returned a slightly higher than expected value for 0.19 kPa.s.L^−1^ but also at 0.49 kPa.s.L^−1^ and 0.69 kPa.s.L^−1^. While the tremoflo deviation from the expected resistance was a parallel shift from the line of identity, the IOS generated a line with slope that deviated significantly from the line of identity. An analysis of the slopes of an identity plot of the measured vs. nominal resistance demonstrated that the tremoflo had a slope of 1.033 ± 0.008 whereas the IOS had a slope of 0.7161 ± 0.015, the slopes were found to be significantly different at p = 0.0030. The IOS was significantly different from the line of identity, p = 0.0028, whereas the slope from the tremoflo was not significantly different from the line of identity, p = 0.057. The deviation from the identity returned by the IOS could explain why the IOS returned lower values for the more severe patients.

Using calibration loads that are in the lower end of the expected range introduces a problem in that even a small error in the calibration probe will be amplified the higher the resistance in the lung gets. In our case it seems like the tremoflo generates higher readings of R5 and lower readings of X5 than IOS. Calibrating at the lower end of the resistance spectrum might introduce an unintentional error in the measurement. This is likely not a big issue if a patient is tested on the same type of device, however, it makes it difficult to compare results obtained from devices calibrated differently, even if the device is of the same make. It would be better if the calibration probes were near the maximally expected range of resistance as this would reduce the risk of amplification or attenuation of the result. The tremoflo factory calibration goes beyond the range of the patients included in this study (personal communication), hence this might be the explanation for why the tremoflo also returned resistance values close to the test loads (Fig. 3). It would have been desirable to use the same calibration loads on both devices, however, this turned out not to be feasible due to the way the devices were manufactured as well as limitations in the software not allowing for alternate calibration routines.

## Conclusion

In analogy with the use in laboratory animals, the use of lung impedance measurements in the clinical setting can provide detailed and precise information about the respiratory function. However, our observations also illustrate that there are potential differences between devices that the user need to be aware of. Our results also suggest that unwittingly to the user a device can be suffering from an erroneous calibration leading to over- or under-estimation of the impedance. It is, however, important to realize that while we discovered differences between the instruments we don’t have any reason to speculate that any of the devices is faulty; we just want to emphasize that under some circumstances it might be difficult to compare data from one instrument with another. It is important to recognize that both devices were able to successfully detect significant changes in impedance in the patient group and would be able to identify abnormalities tied to COPD.

## Data Availability

The datasets used and/or analyzed in the study are available from the corresponding author on reasonable request.
